# Multiple levers for overcoming the recalcitrance of lignocellulosic biomass

**DOI:** 10.1186/s13068-019-1353-7

**Published:** 2019-01-17

**Authors:** Evert K. Holwerda, Robert S. Worthen, Ninad Kothari, Ronald C. Lasky, Brian H. Davison, Chunxiang Fu, Zeng-Yu Wang, Richard A. Dixon, Ajaya K. Biswal, Debra Mohnen, Richard S. Nelson, Holly L. Baxter, Mitra Mazarei, Wellington Muchero, Gerald A. Tuskan, Charles M. Cai, Erica E. Gjersing, Mark F. Davis, Michael E. Himmel, Charles E. Wyman, Paul Gilna, Lee R. Lynd

**Affiliations:** 1 Thayer School of Engineering, Dartmouth College, 14 Engineering drive, Hanover, NH 03755 USA; 2 BioEnergy Science Center, Oak Ridge National Laboratory, Oak Ridge, TN 37831 USA; 3 Department of Chemical and Environmental Engineering and Center for Environmental Research and Technology, Bourns College of Engineering, University of California Riverside, Riverside, CA 92521 USA; 4 Biosciences Division, Oak Ridge National Laboratory, Oak Ridge, TN 37831 USA; 5 Genomics Division, Noble Research Institute, Ardmore, OK 73401 USA; 6 Department of Biological Sciences, University of North Texas, Denton, TX 76203 USA; 7 Complex Carbohydrate Research Center, University of Georgia, Athens, GA 30602 USA; 8 Department of Plant Sciences, University of Tennessee at Knoxville, Knoxville, TN 37996 USA; 9 Bioenergy Science and Technology, National Renewable Energy Laboratory, Golden, CO 80401 USA

**Keywords:** Biomass deconstruction, Recalcitrance, Transgenic switchgrass, *Populus* natural variants, *Clostridium thermocellum*, *Caldicellulosiruptor bescii*, Cotreatment, CELF, Fungal cellulase

## Abstract

**Background:**

The recalcitrance of cellulosic biomass is widely recognized as a key barrier to cost-effective biological processing to fuels and chemicals, but the relative impacts of physical, chemical and genetic interventions to improve biomass processing singly and in combination have yet to be evaluated systematically. Solubilization of plant cell walls can be enhanced by non-biological augmentation including physical cotreatment and thermochemical pretreatment, the choice of biocatalyst, the choice of plant feedstock, genetic engineering of plants, and choosing feedstocks that are less recalcitrant natural variants. A two-tiered combinatoric investigation of lignocellulosic biomass deconstruction was undertaken with three biocatalysts (*Clostridium thermocellum*, *Caldicellulosiruptor bescii,* Novozymes Cellic^®^ Ctec2 and Htec2), three transgenic switchgrass plant lines (COMT, MYB4, GAUT4) and their respective nontransgenic controls, two *Populus* natural variants, and augmentation of biological attack using either mechanical cotreatment or cosolvent-enhanced lignocellulosic fractionation (CELF) pretreatment.

**Results:**

In the absence of augmentation and under the conditions tested, increased total carbohydrate solubilization (TCS) was observed for 8 of the 9 combinations of switchgrass modifications and biocatalysts tested, and statistically significant for five of the combinations. Our results indicate that recalcitrance is not a trait determined by the feedstock only, but instead is coequally determined by the choice of biocatalyst. TCS with *C. thermocellum* was significantly higher than with the other two biocatalysts. Both CELF pretreatment and cotreatment via continuous ball milling enabled TCS in excess of 90%.

**Conclusion:**

Based on our results as well as literature studies, it appears that some form of non-biological augmentation will likely be necessary for the foreseeable future to achieve high TCS for most cellulosic feedstocks. However, our results show that this need not necessarily involve thermochemical processing, and need not necessarily occur prior to biological conversion. Under the conditions tested, the relative magnitude of TCS increase was augmentation > biocatalyst choice > plant choice > plant modification > plant natural variants. In the presence of augmentation, plant modification, plant natural variation, and plant choice exhibited a small, statistically non-significant impact on TCS.

**Electronic supplementary material:**

The online version of this article (10.1186/s13068-019-1353-7) contains supplementary material, which is available to authorized users.

## Background

Whereas the starch-rich endosperm of cereal grain seeds is easily consumed by the emerging seedling, lignocellulose-rich plant cell walls have evolved to be recalcitrant to biological and physical attack. This recalcitrance remains the greatest impediment to low cost biological conversion of lignocellulose to fuels and chemicals [1, 2]. Such conversion is of interest for climate change mitigation [3], improved sustainability of agricultural landscapes [4, 5], and rural economic development [4, 6, 7]. Approaches to overcome the recalcitrance barrier can be grouped into three categories: (1) starting with nature’s best—that is, choosing naturally occurring cellulosic feedstocks that are distinctively amenable to deconstruction and naturally occurring catalysts that are distinctively effective at mediating deconstruction; (2) using biotechnology to improve naturally occurring feedstocks and biocatalysts; and (3) augmentation of biological deconstruction via non-biological means. We refer to these three approaches as ‘recalcitrance levers’. Application of multiple recalcitrance levers is likely beneficial and may be required to process cellulosic biomass at low cost.

Many microorganisms and enzymes have been proposed as agents of plant cell wall deconstruction, although few controlled comparative studies have been reported. Important groups of cellulolytic microorganisms include representatives of the *Bacteria* and *Eukarya* having both aerobic and anaerobic metabolism [8]. Commercial cellulase preparations are derived largely from the aerobic filamentous fungus, *Trichoderma reesei* [9], which has a free cellulase system with a non-complexed architecture [8, 10]. Anaerobic microbes, many of which feature cellulase systems with a complexed architecture [11, 12], have potential to produce biofuels from cellulosic biomass without added enzymes in consolidated bioprocessing (CBP) configurations [13]. Because rates of plant cell wall solubilization are positively correlated with temperature [8], thermophilic cellulolytic microbes such as *Clostridium thermocellum* and *Caldicellulosiruptor bescii* are of particular interest. The multifunctional CelA enzyme of *C. bescii* is one of the most active cellulase components described to date [14]. Whereas *C. thermocellum* produces a multi-enzyme cellulosome complex, *C. bescii* does not [15].

Looking across the diversity of feedstocks and conversion systems, the following trends may be discerned with respect to amenability to biological deconstruction in the absence of thermochemical pretreatment: pre-senescent grass > senescent grass (including most agricultural residues) > woody angiosperms [16, 17]. Achieving high solubilization yields upon enzymatic hydrolysis using fungal cellulase requires more extensive pretreatment for woody gymnosperms than for woody angiosperms [18–20]. Paye et al. [17] compared biomass deconstruction by six biocatalysts acting on mid-season harvested (pre-senescent) switchgrass with no pretreatment other than autoclaving. Total carbohydrate solubilization after 5 days at low solids loading ranged from 24% for *C. bescii* to 65% for *C. thermocellum*. Solubilization values intermediate to these were found for a thermophilic horse manure enrichment, *Clostridium clariflavum*, *Clostridium cellulolyticum*, and simultaneous saccharification and fermentation (SSF) using fungal cellulase (a commercial cellulase mixture of Novozymes Cellic^®^ Ctec2/Htec2). In a subsequent study [13], solubilization of five different lignocellulose feedstocks by *C. thermocellum* cultures was found to be 2- to 4-fold higher than the same commercial cellulase mixture under a broad range of conditions, with the largest differences observed for the most recalcitrant feedstocks.

Modifying cellulosic feedstocks so that they become less recalcitrant has received considerable effort using both targeted genetic engineering and screening of natural variants [21–30]. The BioEnergy Science Center (BESC) has screened over 850 transgenes for overexpression or transgene fragments for knockdown of target gene expression in thousands of *Populus* and switchgrass (*Panicum virgatum* L) transformed lines, and over 1000 natural *Populus* variants for increased amenability to solubilization by fungal cellulase preparations and equal or greater growth yields compared to wild-type controls [21, 27, 29–32]. Promising transgenic switchgrass lines identified in this effort include a plant line in which the gene coding for caffeic acid *O*-methyltransferase (COMT) of the lignin biosynthesis pathway was down-regulated [21], a line overexpressing the MYB4 transcriptional repressor of lignin biosynthesis [22] and a line down-regulated in the expression of a galacturonosyltransferase4 (GAUT4) gene involved in the synthesis of a specific type of pectin polymer [30]. Samples of these transgenic switchgrass lines plus their corresponding controls grown in the field for 2 years [30, 33, 34] became available in quantities sufficient to undertake fermentation studies shortly before initiation of the study reported here. In the same timeframe, *Populus trichocarpa* lines BESC97 and GW9947 have been identified as representative of high and low recalcitrance natural variants, respectively. GW9947 has a mutation in a lignin pathway gene resulting in lowered lignin content [35]. Comparative assessment of the recalcitrance of genetically engineered plants and natural variants using different biocatalysts has not been reported to our knowledge.

For the vast majority of potential cellulosic feedstocks, some form of non-biological augmentation is necessary in order to increase accessibility to biological attack and achieve high solubilization yields. Thermochemical pretreatment of cellulosic biomass to increase carbohydrate solubilization upon subsequent biological processing has been approached using heat and/or added chemicals, and often both, and is widely thought to be necessary in order to biologically process lignocellulosic biomass [36]. Cosolvent-Enhanced Lignocellulose Fractionation (CELF) is a recently proposed thermochemical pretreatment scheme involving exposure to aqueous tetrahydrofuran and dilute acid at elevated temperatures. Near theoretical carbohydrate solubilization yields have been reported using both commercial cellulase preparations and cultures of *C. thermocellum* for several CELF-pretreated feedstocks [37, 38].

Milling partially fermented solids, termed cotreatment, has recently been proposed as an alternative to thermochemical pretreatment for augmenting the capability of biological systems to deconstruct plant biomass [17]. Paye and coworkers found that 5 min of ball milling of residual solids remaining after fermentation of senescent switchgrass by *C. thermocellum* nearly doubled total carbohydrate solubilization (TCS) upon re-inoculation as compared to a control without milling. Greater particle size reduction and solubilization were observed for milling of partially fermented solids as compared to milling unfermented solids. Balch et al. [39] subsequently reported TCS of 88% for senescent switchgrass fermented by *C. thermocellum* in the presence of continuous ball milling.

Building on newly available less recalcitrant feedstocks and recently described non-biological augmentation methods, we report a two-part combinatoric investigation involving three biocatalysts (*C. thermocellum*, *C. bescii*, and fungal cellulase), three transgenic switchgrass plant lines and their respective nontransgenic controls totaling six lines, and two *Populus* natural variants, and augmentation using either mechanical cotreatment or CELF pretreatment. This work was undertaken to gain insight into topics of fundamental and applied significance including the relative and cumulative impact of various recalcitrance levers, the impact of plant modifications on recalcitrance evaluated using different biocatalysts, and the identification of alternative combinations of levers that result in near-complete carbohydrate solubilization.

## Results

Experiments were undertaken aimed at evaluating the impact of multiple potential “levers” by which to impact the recalcitrance of lignocellulosic biomass, both singly and in combination. Levers examined include feedstock natural variants (more or less recalcitrant *P. trichocarpa*), feedstock modification aimed at reducing recalcitrance (three switchgrass lines each with unmodified controls, as described below), choice of feedstock (switchgrass or *Populus*), choice of biocatalyst (*C. thermocellum*, *C. bescii*, or commercial fungal cellulase), and augmentation (CELF pretreatment, cotreatment, and a non-augmented control). Biological replicates were run for all conditions in 0.5 L bioreactors. Since the full combinatoric space involves (8 feedstocks × 3 biocatalysts × 3 augmentations × 2 duplicates) = 144 independent bioreactor experiments, a full factorial design was not practical and a two-tiered experimental design was used. In the first tier, we tested three biocatalysts on three modified switchgrass lines and their non-modified parent lines. In the second tier, we examined the impact of augmentation using the feedstock and biocatalyst that gave the strongest performance in the first tier experiments, and also evaluated two *Populus* natural variants. To assess inherent biocatalytic capability and amenability of feedstocks to deconstruction, we evaluate biomass deconstruction at low solids concentration and in the absence of complicating factors that could arise in industrial processing environments.

### Solubilization of three transgenic switchgrass lines using three biocatalysts

**Fig. 1:**
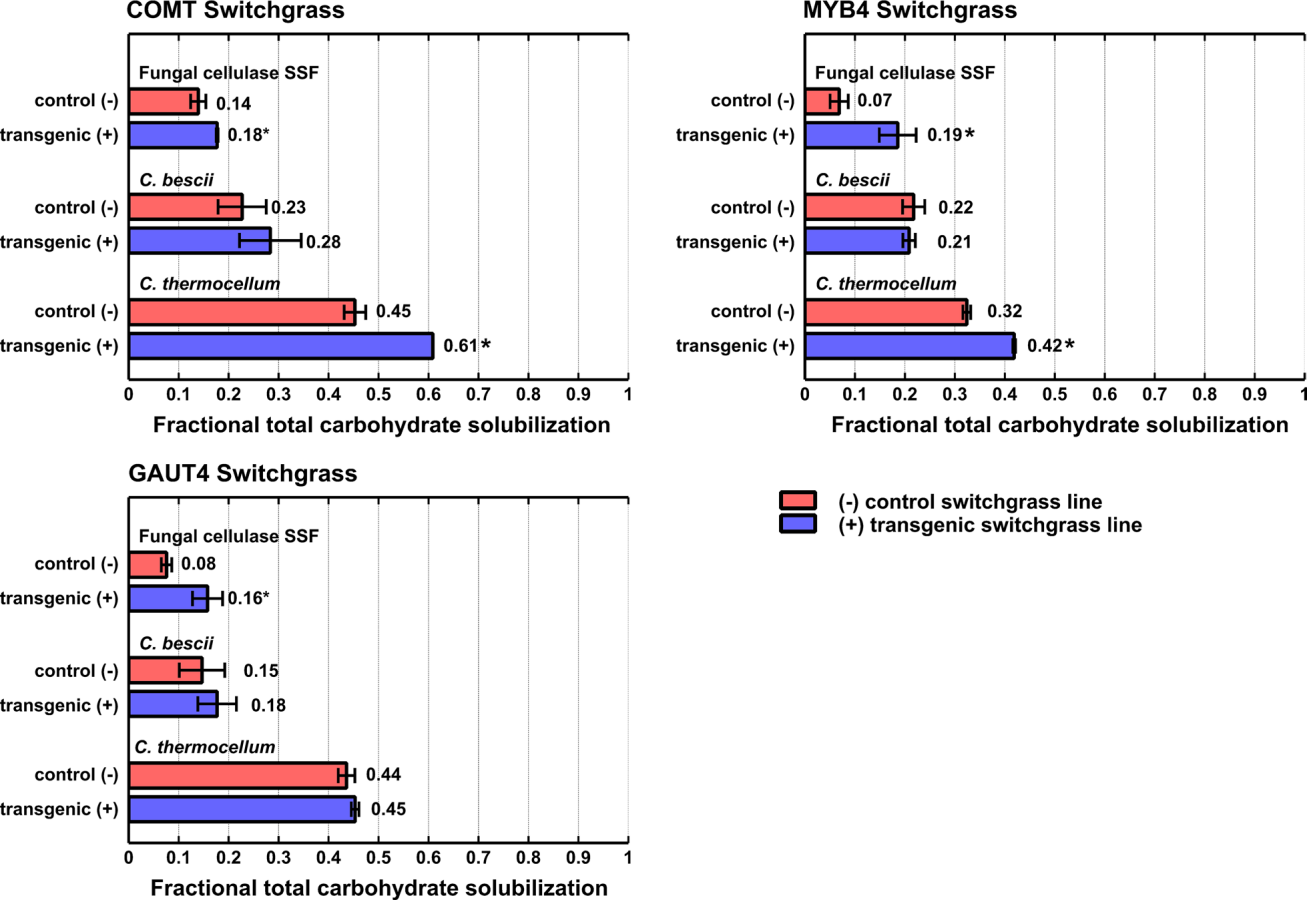
Fractional total carbohydrate solubilization for three transgenic switchgrass lines and their controls mediated by three different biocatalysts. Fungal cellulase was loaded at 5 mg/g solids and in a 9:1 ratio for Ctec2 and Htec2. Red bars show solubilization for the control plant lines (−) and blue bars show solubilization for the transgenic switchgrass lines (+). Initial solids concentrations were based on equal glucan loadings, and fermentations were done in duplicate. Solubilization results are after 120 h of incubation. Error bars represent one standard deviation and are based on biological replicates. Both COMT and MYB4 represent modifications in the lignin pathway, and GAUT4 represents modification in the pectin pathway. An asterisk (*) indicates that the difference in solubilization between transgenic and control plant lines was statistically significant at *p* ≤ 0.05. Details of the statistical analysis are presented in Additional file 2: Table S3

Total carbohydrate solubilization (TCS) was evaluated for three transgenic switchgrass lines, referred to as COMT+, GAUT4+, and MYB4+ as well as their respective nontransgenic control lines, referred to as COMT−, GAUT4−, and MYB4−. Three biocatalysts were used to mediate plant cell wall solubilization: a commercial cellulase preparation (Novozymes Cellic^®^ Ctec2/Htec2, 9:1 ratio at 5 mg/g solids), a culture of *C. thermocellum* DSM 1313, and a culture of *C. bescii* DSM 6725. Tests using the commercial cellulase preparation were carried out in the presence of *Saccharomyces cerevisiae* (strain D_5_A, ATCC 200062), allowing soluble sugars to be consumed as they are formed in a similar manner as occurs for the two cellulolytic cultures. These tests are referred to here as ‘fungal cellulase SSF’. Equivalent TCS with and without yeast, at both 35 and 50°, and at multiple enzyme loadings, has been reported for experiments with added Ctec2 and Htec2 under conditions similar to those reported here [17]. Results are presented in Fig. 1, with numeric values in Additional file 1: Table S1A.

As shown in Fig. 1, TCS ranged from 0.07 to 0.61. In eight of the nine modified switchgrass-biocatalyst combinations, the modified plant line exhibited a higher TCS than the respective unmodified parent line. For *C. thermocellum*, the difference in solubilization between transgenic and control plant lines was statistically significant (*p *≤ 0.05) for COMT and MYB4 but not for GAUT4. For fungal cellulase SSF, TCS was higher and statistically significant for all three transgenic lines compared to their controls (Fig. 1). For *C. bescii*, TCS of transgenic plant lines exceeded respective controls for COMT and GAUT4 but not at a statistically significant level, and there was no increase in solubilization for MYB4 (Additional file 1: Table S1A and Additional file 2: Table S2). For all transgenic plants and their controls, solubilization with *C. thermocellum* was significantly higher than with the other two biocatalysts. Solubilization with *C. bescii* was significantly higher than with fungal cellulase SSF for the MYB4 control, but not for the other transgenic and control lines (Additional file 2: Table S3).

**Fig. 2:**
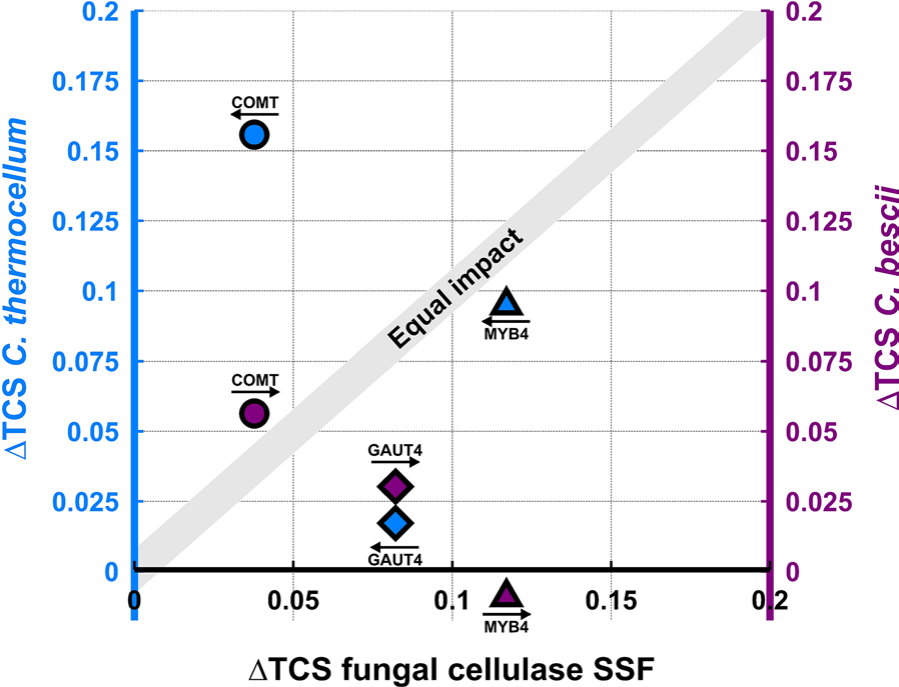
Increased fractional total carbohydrate solubilization (ΔTCS = TCS for transgenic plant lines − TTCS for unmodified controls) for three different plant line pairs and three different biocatalysts. Diamond shaped markers denote GAUT4, circles COMT, and triangles MYB4. ΔTCS for fungal cellulase SSF is on the *x*-axis. ΔTCS is plotted on the left axis for *C. thermocellum*, and on the right axis for *C. bescii*, as indicated by the arrows. The ‘equal impact’ line represents equal increase in solubilization for the different biocatalyst–plant modification combinations. Overall solubilization results are after 120 h of incubation and from duplicate fermentation runs. Both COMT and MYB4 represent modifications in the lignin pathway, and GAUT4 represents modification of the pectin pathway

We hypothesized that TCS enhancement due to plant modifications would be similar for different biocatalysts; e.g., due to changes in substrate accessibility that would be operative for any enzyme system. To visualize the impact of biocatalyst on the difference between transgenic plant lines and their controls, we plotted in Fig. 2 ΔTCS (= TCS for transgenic plant lines − TCS for unmodified control plant lines) for *C. thermocellum* (left vertical axis) and *C. bescii* (right vertical axis) in relation to ΔTCS for fungal cellulase SSF (horizontal axis). For the three plant modifications and three biocatalysts tested, ΔTCS deviates substantially from the equal impact line shown in Fig. 2 for a majority of the data points. Thus, TCS enhancement due to plant modification was found to be highly dependent on the choice of biocatalyst.

### Augmentation of solubilization for three modified switchgrass lines and two Populus natural variants

**Fig. 3:**
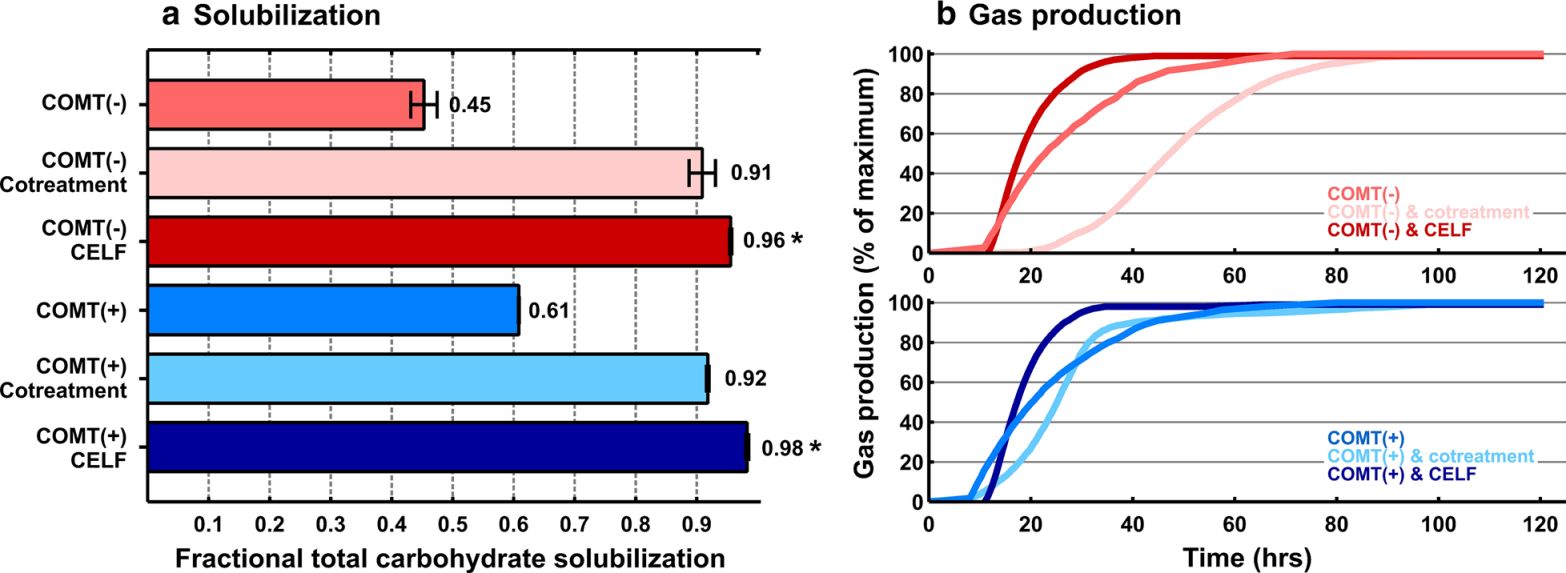
Fractional total carbohydrate solubilization (**a**) and normalized gas production (**b**) for switchgrass fermentation by *C. thermocellum* with and without augmentation by either cotreatment or cosolvent-enhanced lignocellulosic fractionation (CELF). COMT(+) is a transgenic plant line with a down-regulated lignin pathway. COMT(−) is the control plant line. Solubilization results (**a**) are based on equal glucan loadings after 120 h of incubation from duplicate fermentation runs. Error bars for solubilization results represent one standard deviation and are based on biological replicates. For each solubilization bar, one representative gas production data set is shown (**b**); gas production data are a percentage of each respective maximum gas production value after 120 h.. An asterisk (*) indicates that the difference in solubilization between cotreatment and CELF was statistically significant at *p* ≤ 0.05. Details of the statistical analysis are presented in Additional file 2: Table S4

**Fig. 4:**
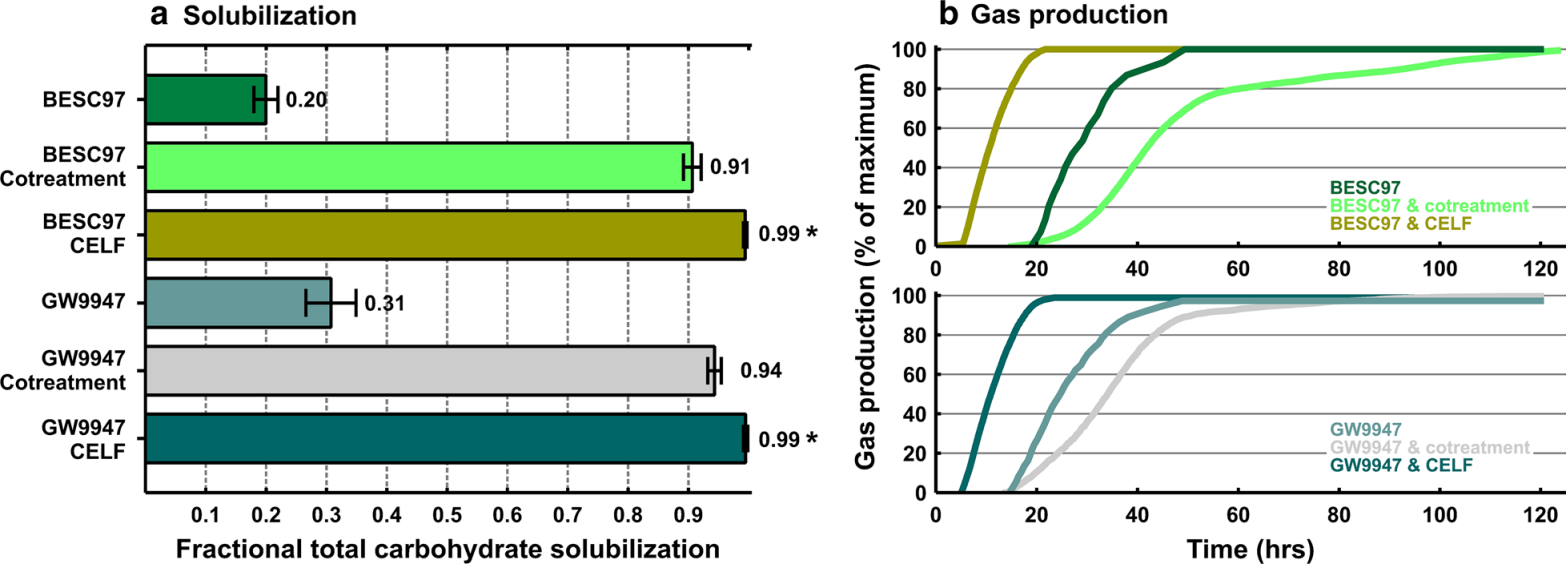
Fractional total carbohydrate solubilization (**a**) and gas production (**b**) for *Populus trichocarpa* fermentation by C*. thermocellum* with and without augmentation. Augmentation categories are cotreatment, and cosolvent-enhanced lignocellulosic fractionation (CELF) and none. Natural variant GW9947 *P. trichocarpa* contains a mutation in the lignin pathway resulting in lower lignin content and BESC97 is a control *P. trichocarpa* line. Solubilization results (**a**) are based on equal glucan loadings after 120 h of incubation from duplicate fermentation runs. Error bars for solubilization results represent one standard deviation and are based on biological replicates. For each solubilization bar, one representative gas production data set is shown (**b**); the gas production data are a percentage of each respective maximum gas production value after 120 h. An asterisk (*) indicates that the difference in solubilization between cotreatment and CELF was statistically significant at *p* ≤ 0.05. Details of the statistical analysis are presented in Additional file 2: Table S4

We next examined the impact of non-biological methods for augmenting biologically mediated solubilization. Two such methods were evaluated: CELF pretreatment and cotreatment via continuous ball milling during fermentation. Tests were carried out using the biocatalyst and feedstock that gave the highest TCS in the experiment depicted in Fig. 1 and the largest benefit of plant modification: *C. thermocellum* and COMT. To see how *C. thermocellum* would perform on a more recalcitrant feedstock, two natural variants of *P. trichocarpa,* GW9947 and BESC97, were also tested. Results are presented in Figs. 3, 4, with numeric values in Additional file 1: Tables S1B, C, Additional file 2: Tables S4 and S5. Figure 3a presents solubilization for COMT transgenic switchgrass (COMT+) and the unmodified control (COMT−), with no augmentation, and augmentation via cotreatment and CELF. Gas production for each feedstock-augmentation combination is presented in Fig. 3b. Data for *P. trichocarpa* natural variants GW9947 and BESC97 are presented in Figs. 4a, b in a similar format.

Augmentation using either CELF pretreatment or cotreatment resulted in TCS in excess of 90% for all tested feedstocks; COMT+, COMT− switchgrass lines, and GW9947 and BESC97 *Populus* variants (Figs. 3a, 4a). Gas production (Figs. 3b, 4b) ceased by the end of the 5-day incubation period, first for CELF-pretreated materials, second for unaugmented feedstock, and last for fermentation with cotreatment.

TCS was higher for CELF pretreatment than for cotreatment for all four plant lines by an average of 0.063 ± 0.018. The difference between CELF and cotreatment was statistically significant for all four feedstocks (Additional file 2: Table S4). In the absence of augmentation, ΔTCS was 0.156 for COMT+ switchgrass relative to its unmodified control COMT− (*p* = 0.005) and 0.108 for BESC97 *Populus* relative to BES9947 (*p* = 0.040). After augmentation by CELF or cotreatment, ΔTCS for COMT + relative to its unmodified parent was reduced by 8.7-fold, ΔTCS for *Populus* GW9947 relative to *Populus* BESC97 was reduced 5.8-fold, and neither of these two ΔTCS values was significant (Additional file 2: Table S5).

### Comparative impact of recalcitrance levers under the conditions tested

**Fig. 5:**
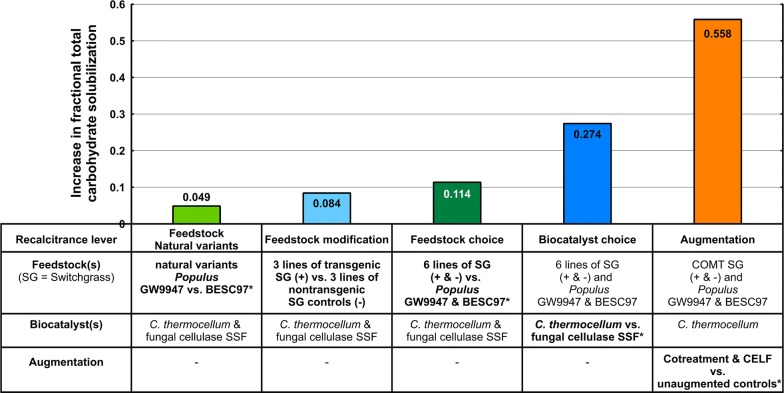
Relative impact of five recalcitrance levers on total carbohydrate solubilization. The increase in total carbohydrate solubilization for each lever in bold is calculated with other levers as indicated. For example, the impact of plant natural variants (*Populus trichocarpa* GW9947 vs BESC97) is calculated using data obtained with both *C. thermocellum* and fungal cellulase SSF without augmentation, the impact of augmentation is calculated using *C. thermocellum* for COMT+ and COMT− switchgrass lines as well GW9947 vs BESC97 Populus lines. *P. trichocarpa* GW9947 has a mutation in the lignin pathway resulting in a lower lignin content; BESC97 is a control without this mutation. Transgenic switchgrass lines COMT and MYB4 have modifications in the lignin pathway, and GAUT4 has a modification in the pectin pathway. Respective nontransgenic controls are included for tests involving the three transgenic switchgrass lines. The recalcitrance lever ‘Augmentation’ includes cotreatment and CELF (Cosolvent-enhanced lignocellulosic fractionation) as well as unaugmented plant controls. Data are calculated from duplicate fermentation runs with equal initial glucan loading. For solubilization data see Additional file 1: Tables S1 A, B, C. Solubilization results are after 120 h of incubation. An asterisk (*) in the tabularized section denotes statistically significant, for full details on the statistical analysis see Additional file 3: Tables and Figures S6–S10

Based on the results for solubilization of switchgrass and *Populus* by *C. thermocellum* (Figs. 1, 3, and 4) and of switchgrass by fungal cellulase SSF (Fig. 1), combined with additional data for *Populus* solubilization by fungal cellulase SSF (Additional file 1: Table S1B), we examined the impact of the recalcitrance levers examined under the conditions tested. As presented in Fig. 5, the relative increase in TCS for the various levers examined under the conditions tested was augmentation > biocatalyst choice > plant choice > plant modification > plant natural variants. The increase in solubilization for each lever was statistically significant except plant modification (Additional file 3: Tables and Figures S6–S10).

## Discussion

Our study is the first known to us to systematically compare in a combinatoric fashion the impact of plant and biocatalyst choice, plant modification, and non-biological augmentation on plant cell wall deconstruction. The extent of TCS enhancement by genetically engineered plants is evaluated for the first time using various biocatalysts, and marked differences are observed. We also show for the first time that high TCS can be achieved with a woody feedstock following cotreatment in lieu of thermochemical pretreatment.

For all plants and conditions tested, including representative woody and herbaceous feedstocks with or without genetic modification in the case of switchgrass and more and less recalcitrant *Populus* natural variants, non-biological augmentation via both CELF pretreatment and cotreatment had a large impact on deconstruction. Based on our results as well as literature studies, it appears that some form of non-biological augmentation will likely be necessary for the foreseeable future in order to achieve high TCS for most cellulosic feedstocks. However, our results show that this need not necessarily involve thermochemical processing and need not necessarily occur prior to biological conversion.

Significant differences were observed in TCS achieved using various biocatalysts. For three different transgenic switchgrass lines and their respective control lines, the order of effectiveness at achieving high TCS was *C. thermocellum *> *C. bescii *> fungal cellulase SSF. *C. thermocellum* was more effective than fungal cellulase SSF for all six switchgrass lines as well as two *Populus* natural variants. The observed impact of biocatalyst choice on biomass deconstruction was smaller than non-biological augmentation, but larger than the plant choice and plant modification or natural variation for the plants and conditions tested. As controlled comparative data become available from this study and others [17], there are increasing indications that biocatalysts differ substantially with respect to their ability to achieve high solubilization yields with lignocellulosic feedstocks. In light of this, we see growing impetus to choose biocatalysts with strong deconstruction capability and to address the biotechnological challenges necessary in order to apply them industrially.

Mechanistic understanding for why complexed cellulosomes achieve higher TCS than the non-complexed cellulase system of *T. reesei* is emerging, although incomplete. The ability of *C. thermocellum* to efficiently attack lignocellulosic fibers with both complexed and non-complexed enzyme systems has been suggested in this context [14, 40]. Moreover, high molecular weight cellulosome complexes display solubilization functionalities unique to these assemblies, such as the ability to splay (and thus access) the ends of cellulose microfibrils, present a diversity of glycoside hydrolases in close proximity to each other, and locate cellulase activity close to the microbial cell [41]. Synergy between enzymes and cellulolytic microbes has been described in the context of lignocellulose solubilization [42] and it is possible that the enzymes present in the commercial cellulase preparation studied would have been more effective at mediating solubilization if they had been tested in the presence of metabolically active cultures of the aerobic fungus that produced them. We focus on metabolically inactive cellulase preparations here because this is the form anticipated for use in industrial bioconversion processes.

In the absence of augmentation, ∆TCS was positive for 8 of the 9 combinations of switchgrass modifications (COMT, MYB4 and GAUT4) and biocatalysts (Fungal cellulase SSF, *C. thermocellum* and *C. bescii*), with this difference statistically significant for five of the combinations. Our results indicate that recalcitrance is not a trait determined by the feedstock only, but instead is coequally determined by the choice of biocatalyst. For the three switchgrass modifications, the average ∆TCS was 0.0790 for fungal cellulase, 0.0258 for *C. bescii*, 0.0893 for *C. thermocellum*, and 0.0647 for all three biocatalysts combined. For the two natural variants of *Populus*, the average ∆TCS (GW9947 minus BESC97) was 0.108 for *C. thermocellum*, − 0.0107 for fungal cellulase, and 0.0485 for both biocatalysts combined. For all the modified and natural variant plant lines tested, the ∆TCS values observed were substantially smaller than those observed for augmentation via CELF or cotreatment (0.558), choice of *C. therm*o*cellum* or fungal cellulase (0.274), and choice of *Populus* or switchgrass (0.114). In the presence of augmentation and under the conditions tested, plant modification, natural variation, and feedstock choice exhibited substantially smaller, statistically non-significant absolute impacts on solubilization.

These observations are supported by controlled experiments with various biocatalysts showing agreement with the literature [17], as well as the experience of investigators with diverse expertise who have collaborated for a decade. The approach taken here to evaluate recalcitrance differs from the recalcitrance assay developed by Selig et al. [43] and used in prior studies by BESC researchers, in being lower throughput, involving several different metabolically active microorganisms in addition to cell-free fungal cellulase preparations, and involving lower fungal cellulase loadings.

Controlled comparative studies of feedstocks and biocatalysts are informative with respect to both fundamental understanding and applications, but have seldom been reported. It is also the case that drawing conclusions from such studies involves considerable nuance and complexity, and that results reported here have limitations. Results from tests made under different conditions would likely be different. In addition, there are many more plant modifications and variants, biocatalysts, and augmentation strategies and conditions that could be tested. The data presented here for both CELF and cotreatment are for a single set of conditions and it is possible that differences between modified or variant plant lines and their more recalcitrant parent or comparative lines might be larger at milder conditions.

Notwithstanding these caveats, our results provide important strategic guidance with respect to overcoming the recalcitrance barrier. The extent of solubilization enhancement by plant genetic engineering was found to be highly dependent upon the biocatalyst used. We showed that solubilization of plant cell walls can be enhanced by non-biological augmentation, the choice of biocatalyst, the choice of plant feedstocks, genetic engineering of plants, and choosing less recalcitrant natural variants. However, the magnitude of enhancement offered by these levers differs markedly under the conditions tested here, with the largest impacts seen for augmentation and the choice of biocatalyst.

## Methods

### Feedstocks

Switchgrass (*Panicum virgatum* L.) transgenic lines and their respective nontransgenic controls were grown in a Knoxville, TN field under USDA APHIS BRS permits as previously described: COMT [33]; MYB4 [34], GAUT4 [29, 30]. *Populus* (*Populus trichocarpa*) samples were provided by the Tuskan laboratory at Oak Ridge National Laboratory, Oak Ridge, TN. Details on origin and compositional analysis for each particular feedstock can be found in the references in the paragraphs discussing each feedstock.

The six switchgrass plant lines (three pairs of two) used in this analysis were second-year field-grown (COMT 2012; MYB4 2013; GAUT4 2014) and were fully senescent upon harvesting. After the first frost in their respective years, the above ground plant biomass was harvested and oven dried at 43 °C for 96 h, and chipped into 3.5–12 cm long and 1.2–3.5 mm diameter wide pieces at the Stewart laboratory, University of Tennessee, Knoxville [33, 34]. Switchgrass feedstock material was then stored in plastic bags indoor under temperature and low-humidity controlled conditions at NREL. Each pair consisted of a control plant line and a transgenic plant line. The three transgenic targets examined are COMT-knockdown [21], MYB4 overexpression [44] and GAUT4-knockdown [30].

COMT switchgrass targeted lignin content and lignin monolignol-composition (S:G) by RNAi of caffeic acid 3-*O*-methyl transferase in the “Alamo” variety [line COMT3(+)] as described in Fu et al. [21]. The corresponding control [line COMT3 (–)] is a null segregant derived from the same parental line [21].

MYB4 switchgrass overexpresses the transcriptional repressor PvMYB4 that results in reduced lignin content (line L8) in the ST1 clone of ‘Alamo’. The control plant (line L7-WT) used was an independent line which had been subjected to the same tissue culture protocol but does not harbor the MYB4 construct [22, 44].

GAUT4 switchgrass is down-regulated in a specific glycosyltransferase in the pectin pathway resulting in lower content of a specific type of pectin [line 4A (+)] in the SA7 clone of ‘Alamo’ [30, 45]. The control plant (line SA7-WT) used was an independent line which had been subjected to the same tissue culture protocol but does not harbor the GAUT4 construct [30].

The natural variants of *Populus trichocarpa* were obtained by felling 4-year-old trees grown in a common garden in Clatskanie, OR. The logs were dried at 70 °C in a forced air oven until constant weight was obtained. Wood cookies were cut from the log with a band saw and milled to + 20 mesh using a Wiley mill (Thomas Scientific, Swedesboro, NJ) [24]. GW9947 has a mutation in a lignin pathway gene resulting lowered lignin content [35], BESC97 served as a reference *Populus* plant line.

### Feedstock preparation

All switchgrass feedstocks were initially cut into 2–4 cm size pieces, milled using a 0.5 mm mill-screen (Retsch mill, Haan, Germany), and sieved through a 0.5 mm sieve-screen. The material not passing the screen was re-milled until all material passed with a maximum of three re-milling sessions. The *Populus* was received at 20 mesh size and milled and sieved at 0.5 mm mill-screen as for switchgrass.

Both types of feedstock were rinsed to remove easily solubilized carbohydrate as described previously [17, 46] followed by drying at room temperature. Carbohydrate content was determined by Quantitative saccharification (QS) [47]. Feedstocks were loaded 5 g glucose equivalent/L loadings, which ranged from 5.2 to 14.7 g/L dry solids for the different feedstock materials tested.

### Cosolvent-enhanced lignocellulosic fractionation (CELF)

CELF pretreatment of unwashed milled switchgrass and *Populus* (0.5 mm particle size, milled as described previously) was performed at 140 °C for 30 min for Switchgrass and at 150 °C for 35 min for poplar, and included a 0.5 wt% sulfuric acid addition in 1:1 (vol) mixture of THF and water. Prior to pretreatment, biomass was soaked overnight in this solution at 10 wt% solids loading with a total reaction mixture of 800 g at 4 °C. A 1 L Hastelloy Parr reactor (236HC series, Parr Instruments Co., Moline, IL) with two stacked pitched blade impellers was used for pretreatment. The heating system was a 4 kW model SBL-2D fluidized sand bath (Techne, Princeton, NJ, USA) and the reactor internal temperature was measured using a K type thermocouple probe (Omega CAIN-18G-18, Omega Engineering Co., Stamford, CT, USA). The reaction was controlled to a desired temperature range (± 2 °C) and quickly submerged in a cold water bath to terminate the reaction. All resulting products were then subjected to vacuum filtration to separate the solids from the liquid. The filtered solids were washed once with THF followed by subsequent washes with DI water until the filtrate pH was tested above 5.5. It was then stored at below 4 °C at > 60% moisture before tests to evaluate solubilization. CELF-pretreated feedstock was not dried before being used in solubilization experiments. The carbohydrate content was determined by measuring the dry weight/water content (MX-50 moisture analyzer A&D, Elk Grove, IL) and performing QS on dried material [47].

### Microorganisms, fungal enzymes, growth medium and culturing conditions

*Saccharomyces cerevisiae* D_5_A (ATCC 200062) was a gift from the National Renewable Energy Laboratory. Inoculation cultures were grown overnight at 37 °C under aerobic conditions in shake-flasks on YPD medium (yeast extract 10 g/L, peptone 20 g/L and dextrose 20 g/L). For cultivation in bioreactors, YP medium was buffered with a 0.05 M citric acid buffer (citric acid monohydrate 20× concentrated brought to pH 4.8 with NaOH) as described in the NREL protocol by Dowe and McMillan [48]. During bioreactor cultivation, the culture was maintained at pH 5.0 with 4 N KOH and kept under anaerobic conditions.

Cellic^®^ CTec2 and HTec2 were a gift from Novozymes A/S (Bagsvaerd, Denmark). CTec2 (4.5 mg protein/g of solid substrate) and Htec2 (0.5 mg protein/g solid substrate) were added to bioreactors at the time of inoculation as described by Paye et al. [17].

*Caldicellulosiruptor bescii* DSM6725 was a gift of the Kelly laboratory at North Carolina State University. The growth medium used for solubilization experiments is modified from DSM 516 medium and contained 0.33 g/L MgCl_2_·6H_2_O, 0.33 g/L KCl, 0.33 NH_4_Cl, 0.14 g/L CaCl_2_·2H_2_O, 84.8 ηg/L Na_2_WO_4_·2H_2_O, 0.1361 g/L KH_2_PO_4_, 0.2787 g/L K_2_HPO_4_, 0.5 g/L yeast extract, 5.0 g/L morpholinopropane sulfonic acid (MOPS), 1.0 g/L l-cysteine HCl·H_2_O, 1.0 g/L NaHCO_3_, 0.25 mg/L resazurin, 0.2 mg/L biotin, 0.2 mg/L folic acid, 1.0 mg/L pyridoxine–HCl (B_6_), 50 ηg/L thiamine-HCl (B_1_), 50 ηg/L riboflavin (B_2_), 50 ηg/L nicotinic acid (B_3_), 50 ηg/L d-Ca-pantothenate, 1ηg/L cobalamin B_12_, 50 ηg/L *P*-amino benzoic acid (PABA), 50 ηg/L lipoic acid, 1.5 mg/L FeCl_2_·4H_2_O, 70 ηg/L ZnCl_2_, 0.1 mg/L MnCl_2_·4H_2_O, 6.0 ηg/L H_-3_BO_3_, 0.19 mg/L CoCl_2_·6H_2_O, 2.0 ηg/L CuCl_2_·2H_2_O, 24.0 ηg/L NiCl_2_·6H_2_O and 36.0 ηg/L Na_2_MoO_4_·2H_2_O.

*Caldicellulosiruptor bescii* inoculum cultures were grown overnight in sealed serum bottles (100 mL working volume) under anaerobic conditions on 5 g/L cellobiose and 5 g glucose equivalent/L switchgrass at 75 °C in 250 mL serum bottles shaking at 200 rpm. Inoculum for bioreactor runs was withdrawn from these bottles via syringe, taking care to minimize introduction of solids from the inoculum.

*Clostridium thermocellum* DSM1313 (*Ruminiclostridium thermocellum*) was obtained from the Deutsche Sammlung von Mikroorganismen and Zellkulturen (DSMZ, Leibnitz, Germany). The medium used was adapted from LC medium [49] and contained: 2.0 g/L KH_2_PO_4_, 3.0 g/L K_2_HPO_4_, 0.1 g/L Na_2_SO_4_, 0.5 g/L urea (CH_4_N_2_O), 0.2 g/L MgCl_2_·6H_2_O, 0.05 g/L CaCl_2_·2H_2_O, 0.0035 g/L FeSO_2_·7H_2_O, 0.025 g/L FeCl_2_·4H_2_O, 1.0 g/L l-cysteine HCl.H_2_O, 20 mg/L pyridoxamine dihydrochloride, 4 mg/L PABA, 2 mg/L d-biotin, 2 mg/L B_12_, 6 mg/L MnCl_2_·4H_2_O, 2.5 mg/L ZnCl_2_, 0.6 mg/L CoCl_2_·6H_2_O, 0.6 mg/L NiCl_2_·6H_2_O, 0.6 mg/L CuSO_4_·5H_2_O, 0.6 mg/L H_3_BO_3_ and 0.6 mg/L Na_2_MoO_4_·2H_2_O.

For inoculation, *C. thermocellum* was grown anaerobically overnight on 5 g/L cellulose (Avicel PH105, FMC Corporation, Philadelphia PA) in 250 mL serum bottles at 100 mL working volume with 5.0 g/L MOPS added for additional buffering.

### Fermentation

Fermentations without cotreatment were done in 0.5 L Sartorius Qplus bioreactors with a working volume of 300 mL. Solid substrates suspended in water were autoclaved for 45 min. Subsequently, the headspace was purged for at least 4 h with ‘ultra pure’ N_2_ gas (Airgas, White River, VT) for both *S. cerevisiae* and *C. bescii* experiments. For *C. thermocellum,* a 20% CO_2_/80% N_2_ gas mixture (Airgas, White River, VT) was used.

For *S. cerevisiae* fermentations, all medium components were concentrated 4×, for *C. bescii* 2× and for *C. thermocellum* medium was prepared as described in Holwerda et al. [49] prior to filter sterilization into the bioreactors. The 2× concentrated medium components for *C. bescii* were purged with a 20% CO_2_/80% N_2_ gas mixture; all other concentrated medium components were purged with N_2_ gas.

The cultivation temperature for *C. thermocellum* was 60 °C and the pH was maintained at 7.0 by addition of 4 N KOH. For *S. cerevisiae* the cultivation temperature was 37 °C and pH was maintained at 5.0 with 4 N KOH, and for *C. bescii* the cultivation temperature was 75 °C and pH = 7.15–7.20 was maintained by addition of 1 N NaOH. All fermentations were inoculated with 5% v/v and incubated for 120 h.

Cotreatment fermentation experiments were done in stainless steel bioreactors with a 1.2 L total bed volume and 600 mL medium working volume as described elsewhere [39]. The reactor was autoclaved for 1 h and purged overnight with a 20% CO_2_/80% N_2_ gas mixture. Temperature was 60 °C and pH was maintained at 7.0 by addition of 2 N KOH via a Sartorius Aplus bioreactor control tower (Sartorius Stedim, Bohemia, New York). Milling was initiated shortly before inoculation.

Gas production (H_2_ and CO_2_ gas combined) was measured using Milligas tip meters (Ritter, Hawthorne, NY) filled with a 0.5 N HCl solution, and data were recorded using Rigamo data-acquisition software provided with the tip meters.

### Measuring feedstock solubilization

Solubilization was based on loss of carbohydrates. The carbohydrate content of the dry feedstock was determined at the start and at the end of the experiment by QS according to the NREL protocol [47] as modified by [50]. After 120 h of incubation, residual material was collected by centrifuge (6 K–10 K × *g*), washed once with water, and dried in a 60 °C oven for at least 96 h after which the final weight was determined. The dried material was then homogenized in a mortar and pestle and prepped for acid hydrolysis (QS). Glucose, xylose and arabinose were determined against known standards using HPLC (Waters, Milford, MA) on a HPX-Aminex 87-H column (Bio-Rad, Hercules, CA) with 5 mM H_2_SO_4_ solution eluent.

Total carbohydrate solubilization (TCS), the fraction of originally present carbohydrate solubilized, was calculated as based on:

$${\text{TCS}} = \left( {{\text{TCi}} - {\text{TCf}}} \right)/{\text{TCi}}$$

where TC is the mass of carbohydrate (on a monomer basis), i denotes initial, and f denotes final. The initial and final mass of carbohydrate was calculated based on the mass fraction of glucose, xylose, and arabinose present in dried solids multiplied by dry weight. ∆TCS for conditions 1 and 2 was calculated using

$$\Delta {\text{TCS}} = {\text{TCS}}_{{{\text{condition}}\;2}} - {\text{TCS}}_{{{\text{condition}}\;1}}$$

### Statistical analysis

Pairwise comparisons with student *t* tests were performed with Microsoft Excel built-in *t* test function and Minitab version 17 (Minitab Inc., State College, PA). Statistical analysis of overall solubilization results and increases in solubilization was done by applying student *t* test, ANOVA and Tukey’s tests using Minitab. Statistical tests and the data used are presented in detail in Additional files 1, 2 and 3. For *t* tests and ANOVA’s, outcomes were considered statistically significant when *p* ≤ 0.05. For Tukey’s tests, a 95% confidence interval was used. For figures showing averages of solubilization results, the data are from duplicate fermentations and error bars represent one standard deviation.

## Additional files

**Additional file 1.** Primary data.

**Additional file 2.** Statistical analysis part A.

**Additional file 3.** Statistical analysis part B

## Abbreviations

COMT: caffeic acid *O*-methyltransferase
MYB4: myeloblastosis 4
GAUT4: galacturonosyltransferase 4
CELF: cosolvent-enhanced lignocellulosic fractionation
TCS: total carbohydrate solubilization
CBP: consolidated bioprocessing
SSF: simultaneous saccharification and fermentation
BESC: The BioEnergy Science Center
DSMZ: Deutsche Sammlung von Mikroorganismen und Zellkulturen
ATCC: American Type Culture Collection
MOPS: morpholinopropane sulfonic acid
QS: quantitative saccharification
