# Correction to: Multiple levers for overcoming the recalcitrance of lignocellulosic biomass

**DOI:** 10.1186/s13068-019-1363-5

**Published:** 2019-02-09

**Authors:** Evert K. Holwerda, Robert S. Worthen, Ninad Kothari, Ronald C. Lasky, Brian H. Davison, Chunxiang Fu, Zeng-Yu Wang, Richard A. Dixon, Ajaya K. Biswal, Debra Mohnen, Richard S. Nelson, Holly L. Baxter, Mitra Mazarei, C. Neal Stewart, Wellington Muchero, Gerald A. Tuskan, Charles M. Cai, Erica E. Gjersing, Mark F. Davis, Michael E. Himmel, Charles E. Wyman, Paul Gilna, Lee R. Lynd

**Affiliations:** 1 Thayer School of Engineering, Dartmouth College, 14 Engineering drive, Hanover, NH 03755 USA; 2 BioEnergy Science Center, Oak Ridge National Laboratory, Oak Ridge, TN 37831 USA; 3 Department of Chemical and Environmental Engineering and Center for Environmental Research and Technology, Bourns College of Engineering, University of California Riverside, Riverside, CA 92521 USA; 4 Biosciences Division, Oak Ridge National Laboratory, Oak Ridge, TN 37831 USA; 5 Genomics Division, Noble Research Institute, Ardmore, OK 73401 USA; 6 Department of Biological Sciences, University of North Texas, Denton, TX 76203 USA; 7 Complex Carbohydrate Research Center, University of Georgia, Athens, GA 30602 USA; 8 Department of Plant Sciences, University of Tennessee at Knoxville, Knoxville, TN 37996 USA; 9 Bioenergy Science and Technology, National Renewable Energy Laboratory, Golden, CO 80401 USA

## Correction to: Biotechnol Biofuels (2019) 12:15 10.1186/s13068-019-1353-7

Following publication of the original article [1], the authors reported the omission of one author name.

C. Neal Stewart Jr. was not included as an author during production process. The publishers apologise for this error. The full author list is given in this correction article. The original article [1] has been updated.
