# Supplementary material for: Multiple levers for overcoming the recalcitrance of lignocellulosic biomass
Source: Biotechnol Biofuels. 2019 Jan 17;12:15. doi: 10.1186/s13068-019-1353-7 (PMC6335785; doi:10.1186/s13068-019-1353-7)
Supplement: Supplementary file 1 — Additional file 1. Primary data. [file 13068_2019_1353_MOESM1_ESM.docx]

**Additional file 1: Primary Data**

***Solubilization data used in figures 1-5***

* The data shown here is rounded at four decimal points, as used in the statistical analysis. The data shown in the Results section is rounded to two decimal points.

*The biocatalyst fungal cellulase SSF is abbreviated to ‘SSF’

*TCS = Total Carbohydrate Solubilization

**Table S1A**: Solubilization data for 6 types of switchgrass (3 pairs of two) with three types of biocatalysts.

|  | **TCS_SSF_** | | **TCS*_C.bescii_*** | | **TCS*_C.thermocellum_*** | |
| --- | --- | --- | --- | --- | --- | --- |
| *Duplicate* | A | B | A | B | A | B |
| COMT- | 0.1285 | 0.1500 | 0.2612 | 0.1930 | 0.4681 | 0.4375 |
| COMT+ | 0.1785 | 0.1756 | 0.3271 | 0.2398 | 0.6090 | 0.6081 |
| MYB4- | 0.0814 | 0.0556 | 0.2332 | 0.2020 | 0.3293 | 0.3183 |
| MYB4+ | 0.2116 | 0.1594 | 0.1996 | 0.2172 | 0.4205 | 0.4168 |
| GAUT4- | 0.0683 | 0.0830 | 0.1789 | 0.1146 | 0.4242 | 0.4477 |
| GAUT4+ | 0.1365 | 0.1791 | 0.2042 | 0.1498 | 0.4477 | 0.4586 |

**Table S1B**: Solubilization data for 2 natural variants of *Populus trichocarpa* (GW447 and BESC97) with Fungal cellulase SSF and *C. thermocellum*.

|  | **TCS_SSF_** | | **TCS*_C.thermocellum_*** | |
| --- | --- | --- | --- | --- |
| *Duplicate* | A | B | A | B |
| BESC97 | 0.1055 | 0.1095 | 0.2137 | 0.1855 |
| GW9947 | 0.1068 | 0.0869 | 0.2781 | 0.3369 |

**Table S1C**: Solubilization data for fermentations with augmentation for *Populus* (BESC97 and GW9947) and COMT-/+ switchgrass. The two types of augmentation used: Co-solvent Enhanced Lignocellulose Fractionation (CELF) and Cotreatment (mechanical feedstock disruption during fermentation).

|  | **TCS_CELF_** | | **TCS_Cotreatment_** | |
| --- | --- | --- | --- | --- |
| *Duplicate* | A | B | A | B |
| COMT- | 0.9570 | 0.9542 | 0.8937 | 0.9243 |
| COMT+ | 0.9809 | 0.9843 | 0.9196 | 0.9165 |
| BESC97 | 0.9962 | 0.9920 | 0.9168 | 0.8961 |
| GW9947 | 0.9920 | 0.9973 | 0.9511 | 0.9354 |
