# Supplementary material for: Multiple levers for overcoming the recalcitrance of lignocellulosic biomass
Source: Biotechnol Biofuels. 2019 Jan 17;12:15. doi: 10.1186/s13068-019-1353-7 (PMC6335785; doi:10.1186/s13068-019-1353-7)
Supplement: Supplementary file 2 — Additional file 2. Statistical analysis part A. [file 13068_2019_1353_MOESM2_ESM.docx]

**Additional file 2: Statistical Analysis part A**

***Data and statistical analysis for figures 1-4***

*The biocatalyst *fungal cellulase SSF* is abbreviated to ‘SSF’.

*Solubilization data used in the statistical analysis were at four decimal points (Supplementary Table 1A-C), results of the statistical analysis described here are shown in two (*t*-value) or three (p-value) decimal points.

*All *t*-tests were considered one-tailed, unpaired and homoscedastic.

**Table S2**: Results of *t*-tests for modified (+) vs. unmodified (-) switchgrass for all three biocatalysts. Hypothesis Testing: H­_o_: **_+ _**= **_−_**, H­_1_: **_+_**_ _ **_−_**. P values reflect the probability that rejecting the null hypothesis (equal solubilization for modified and unmodified lines) and accepting the alternative hypothesis (modified solubilization is greater than solubilization for unmodified lines) is not correct.

| **DoF: 2** | **SSF** | | ***C. bescii*** | | ***C. thermocellum*** | |
| --- | --- | --- | --- | --- | --- | --- |
| Modification | **t-value** | **p-value** | **t-value** | **p-value** | **t-value** | **p-value** |
| COMT | 3.48 | 0.037 | 1.02 | 0.208 | 10.18 | 0.005 |
| MYB4 | 4.02 | 0.028 | -0.51 | 0.671 | 16.35 | 0.002 |
| GAUT4 | 3.65 | 0.034 | 0.72 | 0.274 | 1.33 | 0.158 |

**Table S3**: Results of *t*-tests for average solubilization for *C. thermocellum* vs SSF and *C. bescii*, and *C. bescii* vs SSF*.* P-values reflect the probability that the null hypothesis (equal solubilization for *C. thermocellum* and either fungal cellulase SSF or *C. bescii* and equal solubilization for fungal cellulase and *C.bescii* ) is correct.

| **DoF: 2** | ***C. thermocellum* vs. SSF** | | ***C. thermocellum* vs. *C. bescii*** | | ***C. bescii* vs. SSF** | |
| --- | --- | --- | --- | --- | --- | --- |
| Modification | **t-value** | **p-value** | **t-value** | **p-value** | **t-value** | **p-value** |
| COMT- | 16.77 | 0.002 | 6.04 | 0.013 | 2.46 | 0.067 |
| COMT+ | 284.21 | 0.000 | 7.45 | 0.009 | 2.44 | 0.124 |
| MYB4- | 18.21 | 0.002 | 6.42 | 0.012 | 7.37 | 0.009 |
| MYB4+ | 8.91 | 0.006 | 23.38 | 0.001 | 0.82 | 0.216 |
| GAUT4- | 26.00 | 0.001 | 8.45 | 0.007 | 2.16 | 0.082 |
| GAUT4+ | 13.43 | 0.003 | 9.95 | 0.005 | 0.56 | 0.317 |

**Table S4**: Increase in TCS between CELF and cotreatment for solubilization with *C. thermocellum* on COMT modified switchgrass and natural variants of *P. trichocarpa*. P-values reflect the probability that the null hypothesis (equal solubilization for CELF and cotreatment) is correct.

| **DoF: 2** | ***CELF* vs. cotreatment** | | |
| --- | --- | --- | --- |
| Modification/natural variants | **ΔTCS** | **t-value** | **p-value** |
| COMT- | 0.0466 | 3.03 | 0.047 |
| COMT+ | 0.0646 | 28.06 | 0.001 |
| BESC97 | 0.0876 | 8.30 | 0.007 |
| GW9947 | 0.0514 | 6.20 | 0.013 |
| Average | 0.6255±0.018 |  |  |

**Table S5**: Increase in TCS for the COMT modification in switchgrass and the natural variants in *P. trichocarpa* with *C. thermocellum* for no augmentation and with augmentation (CELF and cotreatment are averaged). P-values reflect the probability that the null hypothesis (equal solubilization for the COMT modification or natural variants on *Populus*) is correct.

|  | **Without augmentation**  **(DoF:2)** | | | **With augmentation; average of CELF and cotreatment (DoF:6)** | | | **ΔTCS^a^/ΔTCS^b^** |
| --- | --- | --- | --- | --- | --- | --- | --- |
| Feedstock | **ΔTCS^a^** | **t-value** | **p-value** | **ΔTCS^b^** | **t-value** | **p-value** |  |
| Switchgrass COMT- vs. COMT+ | 0.1558 | 10.18 | 0.005 | 0.0180 | 0.76 | 0.239 | 8.6556 |
| *P.trichocarpa*  BESC97 vs. GW9947 | 0.1079 | 3.31 | 0.040 | 0.0187 | 0.63 | 0.277 | 5.7700 |
