# Supplementary material for: Multiple levers for overcoming the recalcitrance of lignocellulosic biomass
Source: Biotechnol Biofuels. 2019 Jan 17;12:15. doi: 10.1186/s13068-019-1353-7 (PMC6335785; doi:10.1186/s13068-019-1353-7)
Supplement: Supplementary file 3 — Additional file 3. Statistical analysis part B [file 13068_2019_1353_MOESM3_ESM.docx]

**Additional file 3: Statistical Analysis part B**

***Data and statistical analysis used in figure 5***


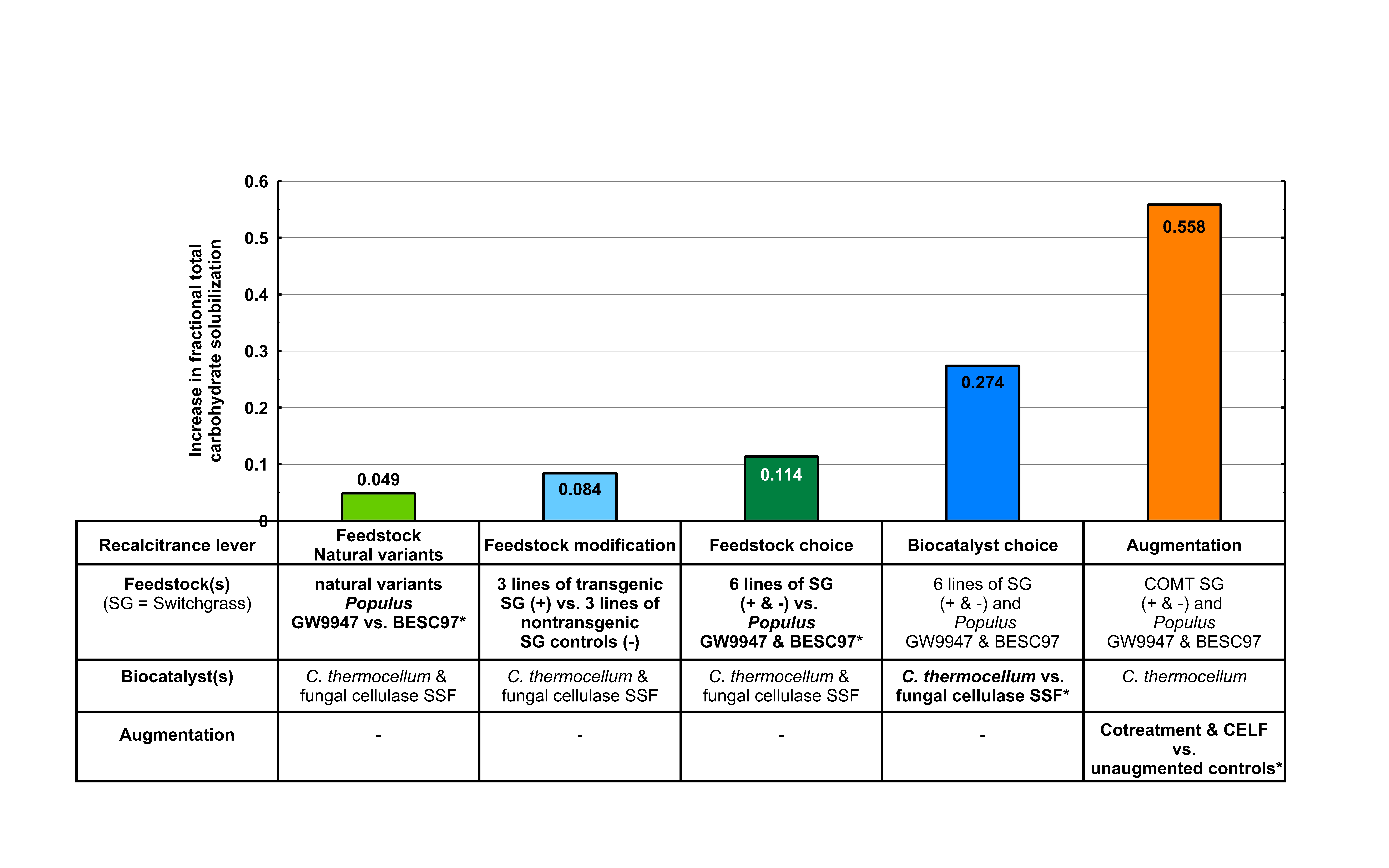


*Figure 5 as shown in the manuscript. An asterisk (*) in the table denotes statistically significant.*

*Solubilization data used in the statistical analysis were at four decimal points (from Supplemental Information S1).

***Feedstock natural variants (bar 1)***

**Table S6A&B**: Increase in solubilization (**ΔTCS**) for 2 natural variants of *Populus* (GW9947 and BESC97) with Fungal cellulase SSF and *C. thermocellum*. Using values from table S1B.

1. Primary and calculated data.

|  | **BESC97 TCS_avg_** | **GW9947 TCS_avg_** | **ΔTCS_avg_** |  |
| --- | --- | --- | --- | --- |
| **SSF** | 0.1075 | 0.0968 | -0.0107 | **ΔTCS*_Populus_* _SSF_** |
| ***C. thermocellum*** | 0.1996 | 0.3075 | 0.1079 | **ΔTCS*_Populus C. thermocellum_*** |
|  |  |  |  |  |
| **Overall average increase solubilization for natural variants of *Populus*** | | | **0.0486** | **ΔTCS_natural variants_ *_Populus_*** |

1. Analysis of variance.

| Analysis of variance | | | | | |
| --- | --- | --- | --- | --- | --- |
| Source | DF | Adj SS | Adj MS | F-Value | P-Value |
| Feedstock | 1 | 0.004725 | 0.004725 | 8.10 | 0.047 |
| Catalysts | 1 | 0.045823 | 0.045823 | 78.56 | 0.001 |
| Feedstock*Catalysts | 1 | 0.007024 | 0.007024 | 12.04 | 0.26 |
| Error | 5 | 0.002333 | 0.000583 |  |  |
| Total | 7 | 0.059905 |  |  |  |


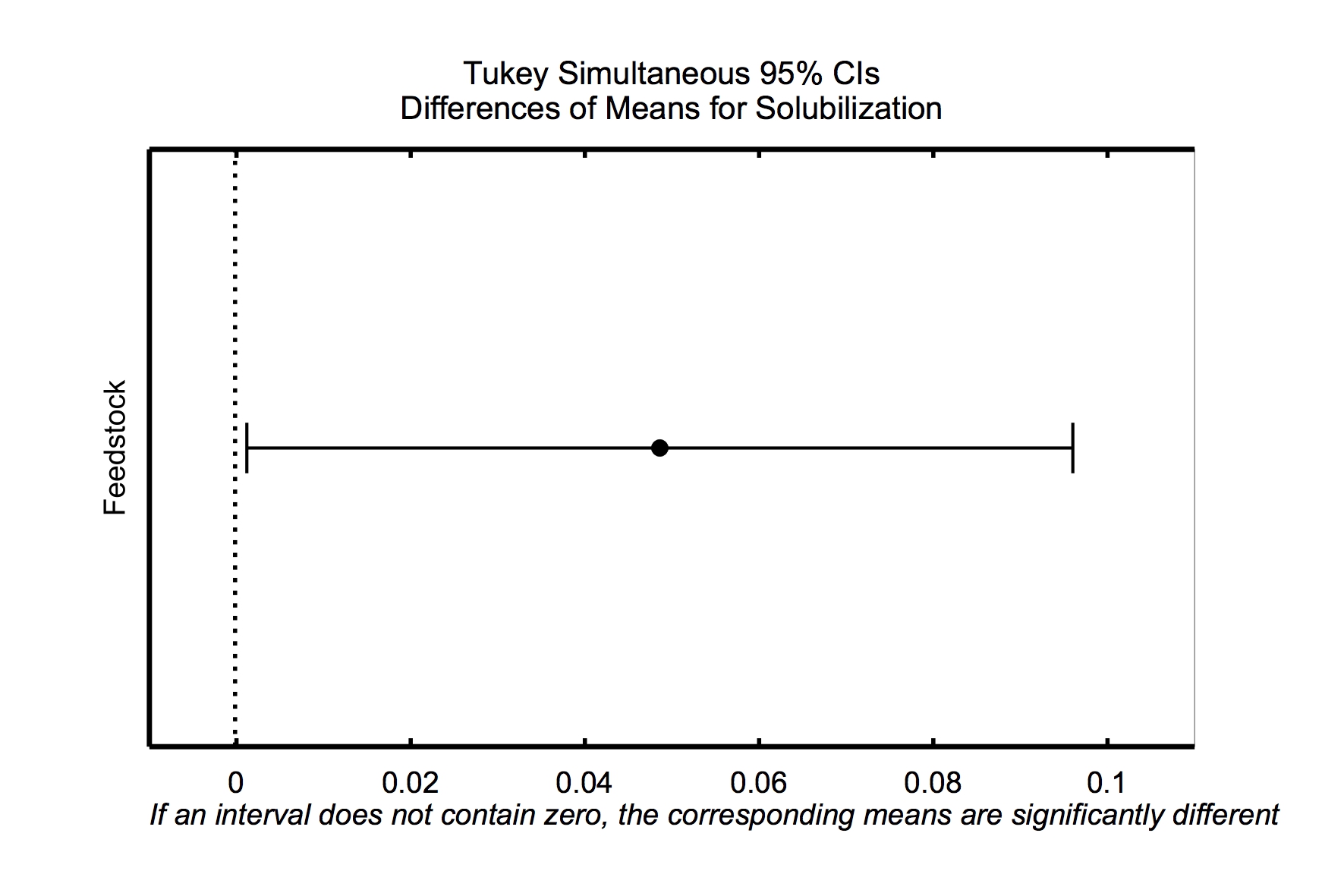


***Figure S6C****: Tukey analysis of the increase in solubilization for bar 1.****Feedstock modification (bar 2)***

**Table S7A&B**:Increase in solubilization (**ΔTCS**) for parent vs. modified plant line for 6 types of switchgrass (3 pairs of two) with Fungal cellulase SSF and *C. thermocellum*. Using values from table S1A.

1. Primary and calculated data.

| **COMT** | **TCS**_COMT- avg_ | _stdev_ | **TCS**_COMT+avg_ | _stdev_ | **ΔTCS_COMT_** |  |
| --- | --- | --- | --- | --- | --- | --- |
| SSF | 0.1393 | 0.0152 | 0.1770 | 0.0021 | 0.0378 | **ΔTCS_COMT SSF_** |
| *C. thermocellum* | 0.4528 | 0.0216 | 0.6085 | 0.0006 | 0.1558 | **ΔTCS_COMT_ *_C. thermocellum_*** |
|  |  |  |  |  |  |  |
| **MYB4** | **TCS**_MYB4- avg_ | _stdev_ | **TCS**_MYB4+avg_ | _stdev_ | **ΔTCS_MYB4_** |  |
| SSF | 0.0685 | 0.0182 | 0.1855 | 0.0369 | 0.1170 | **ΔTCS_MYB4 SSF_** |
| *C. thermocellum* | 0.3238 | 0.0078 | 0.4186 | 0.0026 | 0.0948 | **ΔTCS_MYB4_ *_C. thermocellum_*** |
|  |  |  |  |  |  |  |
| **GAUT4** | **TCS_GAUT4-_** _avg_ | _stdev_ | **TCS_GAUT4+_** _avg_ | _stdev_ | **ΔTCS_GAUT4_** |  |
| SSF | 0.0757 | 0.0104 | 0.1578 | 0.0301 | 0.0821 | **ΔTCS_GAUT4 SSF_** |
| *C. thermocellum* | 0.4359 | 0.0166 | 0.4531 | 0.0077 | 0.0172 | **ΔTCS_GAUT4_ *_C. thermocellum_*** |
|  |  |  |  |  |  |  |
| **Overall average increase solubilization due to modification**  **ΔTCS_COMT&MYB4&GAUT4,_ *_C. thermocellum_* _& SSF_** | | | | | **0.0841** | **ΔTCS_modification_** |

1. Two sample t-test

| t-test for difference: difference = 0 vs (>0)  Difference = μ (modified)- μ (non-modified), Estimate for difference: 0.0841 | | | | | | | | |
| --- | --- | --- | --- | --- | --- | --- | --- | --- |
| Variable | N | Mean | StDev | SE mean | 95% lower bound | T | P | DF |
| Modified | 12 | 0.333 | 0.179 | 0.052 | -0.0381 | 1.18 | 0.125 | 21 |
| Non-modified | 12 | 0.249 | 0.169 | 0.049 |  |  |  |  |

***Feedstock choice (bar 3)***

**Table S8ABC**: Increase in solubilization (**ΔTCS**) between Fungal cellulase SSF and *C. thermocellum* for 6 types of switchgrass (3 pairs of two) and 2 natural variants of *Populus*. Using data from tables S1A&B.

1. Primary and calculated data.

|  | **SSF TCS_avg_** | ***C. thermocellum TC*S_avg_** | **TCS_Total, avg_** |  |
| --- | --- | --- | --- | --- |
| **COMT** | 0.1581 | 0.5306 | 0.3444 | **TCS**_COMT, avg_ |
| **MYB4** | 0.1270 | 0.3712 | 0.2491 | **TCS**_MYB4, avg_ |
| **GAUT4** | 0.1167 | 0.4445 | 0.2806 | **TCS**_GAUT4, avg_ |
| **BESC97** | 0.1075 | 0.1996 | 0.1535 | **TCS**_BESC97, avg_ |
| **GW9947** | 0.0968 | 0.3075 | 0.2021 | **TCS**_GW9947, avg_ |
|  |  |  |  |  |
| Overall average solubilization for switchgrass | | | 0.2914 | **TCS**_SG, avg_ |
| Overall average solubilization for *Populus* | | | 0.1778 | **TCS***_Populus_*_, avg_ |
| **Overall average increase solubilization due to feedstock choice** | | | **0.1135** | **ΔTCS_SG-_*_Populus_*** |

1. Analysis of variance

| **Analysis of variance** | | | | | |
| --- | --- | --- | --- | --- | --- |
| Source | DF | Adj SS | Adj MS | F-Value | P-Value |
| Catalyst | 1 | 0.600477 | 0.600477 | 1562.74 | 0.000 |
| Feedstock | 7 | 0.165869 | 0.023696 | 61.67 | 0.000 |
| Catalyst*Feedstock | 7 | 0.073389 | 0.010484 | 27.28 | 0.000 |
| Error | 16 | 0.006148 | 0.000384 |  |  |
| Total | 31 | 0.845883 |  |  |  |

1. Student t-test

| **t-test for difference: difference = 0 vs (>0)**  Difference = μ (Switchgrass)- μ (Poplar), Estimate for difference: 0.1135 | | | | | | | | |
| --- | --- | --- | --- | --- | --- | --- | --- | --- |
| **Variable** | **N** | **Mean** | **StDev** | **SE mean** | **95% lower bound** | **T** | **P** | **DF** |
| Switchgrass | 24 | 0.291 | 0.176 | 0.036 | 0.0304 | 2.34 | 0.014 | 23 |
| Poplar | 8 | 0.1778 | 0.0925 | 0.033 |  |  |  |  |

***Biocatalyst choice (bar 4)***

**Table S9AB**: Increase in solubilization (**ΔTCS**) between 6 types of switchgrass (3 pairs of two) and 2 natural variants of *Populus* with Fungal cellulase SSF and *C. thermocellum*. Using data from table S1A&B.

1. Primary and calculated data.

|  | **TCS**_COMT, avg_ | **TCS**_MYB4, avg_ | **TCS**_GAUT4, avg_ | **TCS**_BESC97, avg_ | **TCS**_GW9947, avg_ | **TCS_Total, avg_** |  |
| --- | --- | --- | --- | --- | --- | --- | --- |
| SSF | 0.1581 | 0.1270 | 0.1167 | 0.1075 | 0.0968 | 0.1260 | **TCS_SSF Total, avg_** |
| *C. thermocellum* | 0.5306 | 0.3712 | 0.4445 | 0.1996 | 0.3075 | 0.4000 | **TCS*_C. thermocellum Total, avg_*** |
| **Overall average increase solubilization due to biocatalyst choice** | | | | | | **0.2740** | **ΔTCS_Ctherm-SSF_** |

1. Analysis of variance

| **Analysis of variance** | | | | | |
| --- | --- | --- | --- | --- | --- |
| **Source** | **DF** | **Adj SS** | **Adj MS** | **F-Value** | **P-Value** |
| Model | 15 | 0.839735 | 0.055982 | 145.69 | 0.000 |
| Linear | 8 | 0.766346 | 0.095793 | 249.30 | 0.000 |
| Catalyst | 1 | 0.600477 | 0.600477 | 1562.74 | 0.000 |
| Feedstock | 7 | 0.165869 | 0.023696 | 61.67 | 0.000 |
| 2-Way interactions | 7 | 0.073389 | 0.010484 | 27.28 | 0.000 |
| Catalyst*Feedstock | 7 | 0.073389 | 0.010484 | 27.28 | 0.000 |
| Error | 16 | 0.006148 | 0.000384 |  |  |
| Total | 31 | 0.845883 |  |  |  |


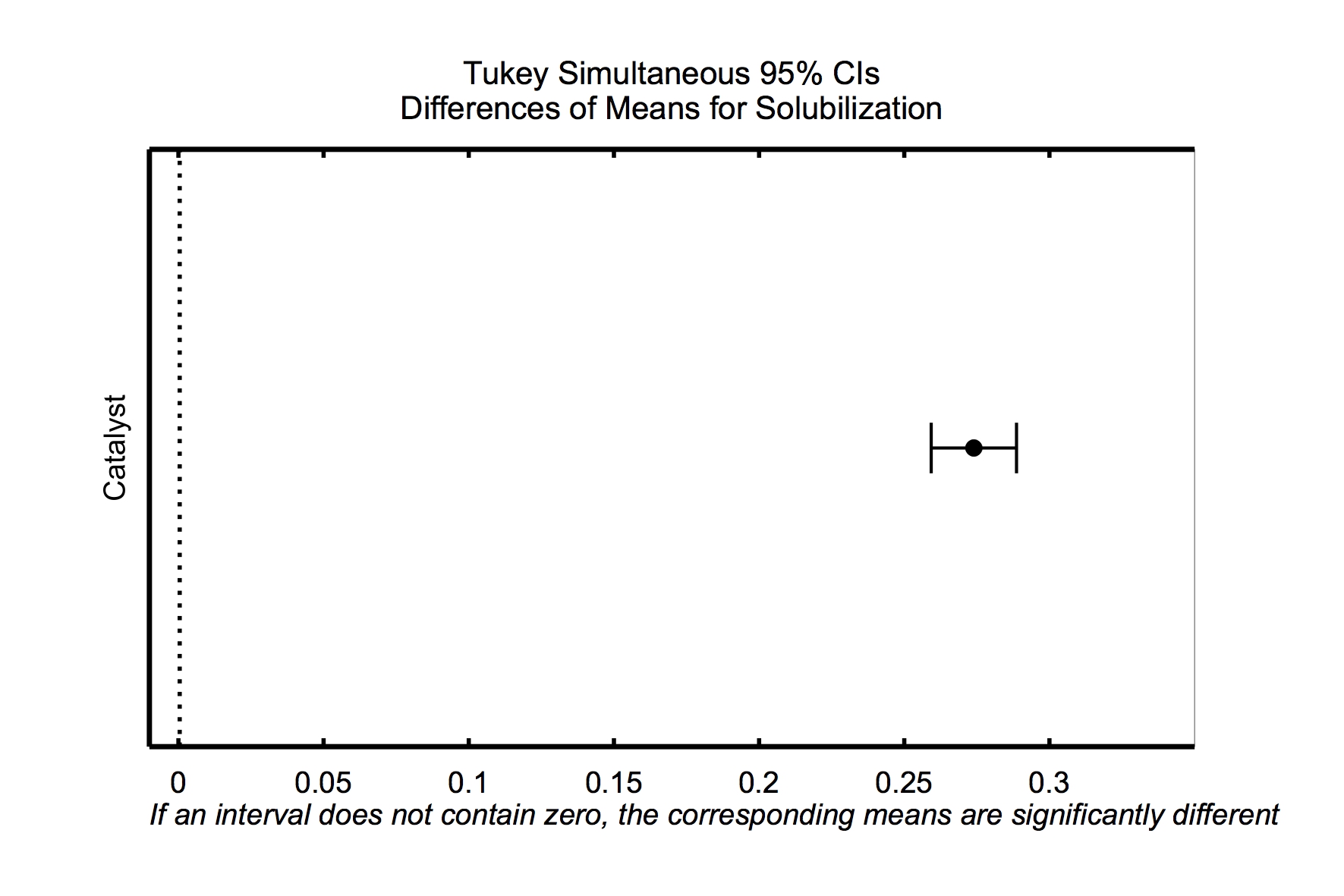


***Figure S9C****: Tukey analysis of the increase in solubilization for bar 4.*

***Augmentation (bar 5)***

**Table S10A&B**: Increase in solubilization (**ΔTCS**) of augmentation (CELF and Cotreatment) vs no augmentation (None) for 2 type of switchgrass (COMT) and 2 natural variants of *Populus* with *C. thermocellum*. Using data from table S1A&B&C.

1. Analysis of variance

|  | **None TCS_avg_** |  | **CELF TCS_avg_** | **ΔTC_CELF-None_** | **Cotreatment TCS_avg_** | **ΔTC_Cotreatment-None_** |
| --- | --- | --- | --- | --- | --- | --- |
| **COMT-** | 0.4528 |  | 0.9556 | 0.5028 | 0.9090 | 0.4563 |
| **COMT+** | 0.6085 |  | 0.9826 | 0.3741 | 0.9181 | 0.3095 |
| **BESC97** | 0.1996 |  | 0.9941 | 0.7945 | 0.9065 | 0.7069 |
| **GW9947** | 0.3075 |  | 0.9947 | 0.6872 | 0.9432 | 0.6358 |
|  |  |  |  |  |  |  |
| Average increase solubilization per augmentation | | | **ΔTCS_CELF_** _avg_ | 0.5897 | **ΔTCS_cotreatment_** _avg_ | 0.5271 |
| Overall average increase solubilization augmentation *Populus* | | | **ΔTCS** _augmentation overall_ _avg_ | | 0.5584 | |

1. Analysis of variance

| **Analysis of variance** | | | | | |
| --- | --- | --- | --- | --- | --- |
| **Source** | **DF** | **Adj SS** | **Adj MS** | **F-Value** | **P-Value** |
| Feedstock | 3 | 0.18020 | 0.060067 | 159.34 | 0.000 |
| Augmentation | 3 | 2.50998 | 0.836660 | 2219.43 | 0.000 |
| Feedstock*Augmentation | 9 | 0.20246 | 0.022496 | 59.67 | 0.000 |
| Error | 16 | 0.00603 | 0.000377 |  |  |
| Total | 31 | 2.89868 |  |  |  |
